# The Stress Response of Aphids to the Accumulation of Heavy Metals Along *Vicia faba* L. Under Cadmium Treatment

**DOI:** 10.3390/insects15120999

**Published:** 2024-12-16

**Authors:** Yexin Xie, Shasha Wang, Sijing Wan, Liya Chen, Qintian Shen, Keting Zhao, Shiyu Tao, Wenjing Zhou, Xinyi Zhang, Xiaoling Tan, Binghua Xie, Bin Tang

**Affiliations:** 1 College of Life and Environmental Sciences, Hangzhou Normal University, Hangzhou 311121, China; xyx7202023@126.com (Y.X.); wangshasha1997@163.com (S.W.); wsjw9898@163.com (S.W.); liya1291803217@163.com (L.C.); 2023210301085@stu.hznu.edu.cn (Q.S.); 2023210301194@stu.hznu.edu.cn (K.Z.); 13858300864@163.com (S.T.); 2023210301184@stu.hznu.edu.cn (W.Z.); 2023210301033@stu.hznu.edu.cn (X.Z.); 2 State Key Laboratory for Biology of Plant Diseases and Insect Pests, Institute of Plant Protection, Chinese Academy of Agricultural Sciences, Beijing 100193, China; tanxiaoling2010@163.com; 3 Zhongyuan Research Center, Chinese Academy of Agricultural Sciences, Xinxiang 453500, China

**Keywords:** cadmium, food chain, *Vicia faba* L., *Megoura crassicauda*, bioaccumulation, energy metabolism

## Abstract

Due to the intensification of human activities, the ecosystems are being polluted by heavy metals. The pollution of heavy metals in agricultural systems has become a serious issue of global concern. This study detected the bioaccumulation of cadmium (Cd) in broad beans and aphids through continuous exposure to varying concentrations of Cd pollution (0, 3.125, 6.25, 12.5, 25, 50 mg/L) and subsequently examined its effects on aphid energy metabolism and reproductive ability. The results showed that Cd can be transmitted and accumulated between *Vicia faba* L. and aphids along the food chain, and the amount of accumulation was related to the Cd treatment concentration. Quantitative real-time PCR results showed that the expression levels of trehalase (*TRE*) and trehalose-6-phosphate synthase (*TPS*) in F1 were significantly upregulated, and those of vitellogenin (*Vg*) were varied across the five generations of aphids after Cd treatment, which were up-regulated, and others down-regulated. Compared with the control group, the glycogen content and two types of trehalase activities of the first-generation Cd-treatment aphids were decreased, while trehalose content increased; there was no significant change in the carbohydrate content and trehalase activity of the fourth and fifth generations of aphids. In addition, the reproduction of female aphids was inhibited. This research is helpful for studying the toxic effects of heavy metals on insects and the adaptation mechanisms of insects to extreme environments. It also provides a theoretical basis for further exploring the molecular mechanisms of Cd homeostasis in plants and insects under Cd stress.

## Simple Summary

The pollution of heavy metals in agriculture has attracted increasing global attention. Heavy metals not only destroy the structure and function of soil but also transfer and accumulate among nutrient levels along the food chain, affecting the growth and development of plants and animals. In this study, we found that Cd could be transferred and accumulated along the food chain between *Vicia faba* L. and aphids. The carbohydrate content and the expression levels of trehalase (*TRE*), trehalose-6-phosphate synthase (*TPS*), and vitellogenin (*Vg*) were up-regulated or down-regulated over 5 generations of aphids after Cd treatment. These findings provide a theoretical basis for studying the toxic effects of heavy metals on phytophagous insects and their resistance mechanisms.

## 1. Introduction

In recent years, the global issue of heavy metal and persistent organic pollutant pollution has received increasing attention [1,2]. Heavy metals are a group of metal elements with a density greater than 5 g/cm^3^ and an atomic mass exceeding the mass of calcium (MW = 40) [3]. Agricultural soils contaminated with metals may include two types of heavy metals. Toxic elements such as cadmium (Cd), lead (Pb), and mercury (Hg) exhibit high toxicity to living cells even at extremely low concentrations [4,5]. Other essential microelements, such as iron (Fe), zinc (Zn), nickel (Ni), copper (Cu), and manganese (Mn), play a key role in several metabolic reactions in plants, and therefore have beneficial effects on plant growth at optimal concentrations [6,7,8,9]. However, when they are excessively present, they have harmful effects on plants [10]. Heavy metal pollution is mainly caused by human factors such as metal smelting, mine development, transportation, the use of pesticides and fertilizers, and sewage irrigation, as well as natural factors such as mudslides and volcanic eruptions [11]. Heavy metals in the environment cannot be degraded by soil microorganisms, but only change in price and form, so heavy metals can exist in the soil for a long time [12].

Cadmium is highly soluble and readily available and rapidly absorbed by plants and introduced into the food chain, and awareness of its pollution in agroecosystems is increasing [13]. It is toxic to all organisms, including plants, animals, and even humans [14,15]. Heavy metal contaminants will infiltrate into groundwater and soil, bioaccumulate in the food webs, and have long-term effects on biological communities, leading to local adaptation and affecting their genetic structures [16]. Since soil is the main growth substrate of plants, heavy metals accumulated in woodland or farmland can be absorbed by roots through plant transpiration and heavy metal diffusion, then transported to the overground part, and eventually accumulated in various parts of the plant [11]. The average bio-enrichment coefficient of heavy metals in crops was Cd > Zn > Cu > Ni > Hg > Cr > Pb [17]. As a non-threshold toxin, Cd can cause serious harm to ecosystems even at relatively low concentrations, highlighting its adverse effects on organisms living in Cd-contaminated areas [18,19,20]. In addition, Cd has high accumulation and mobility in the soil–plant system, which exacerbates its ecological toxicity effects [21]. Heavy metals accumulated in agricultural crops not only affect crop growth and yield but also their accumulation and transfer along the food chain, which may have a certain impact on organisms at higher trophic levels [2,22,23].

Phytophagous insects can be exposed to heavy metals in various ways, mainly including “soil-plant-insect” food chain transmission, artificial feed addition, field exposure, and direct injection in vitro [24]. Phytophagous insects are major consumers of plants and play a vital functional role in transporting toxic metals and energy through terrestrial ecosystems [25]. Current studies have shown that heavy metals could be transferred through the food chain to phytophagous insects and had various chronic toxicological effects on their physiology and growth [26]. Dietary intake is the main source of metal accumulation in arthropods [27]. Research has found that the metal tolerance of *Spodoptera exigua* exposed to a cadmium-contaminated diet for one or many (33 or 61) generations built over time [28]. In the face of heavy metal stress, the population growth of herbivorous insects has changed. For example, in *Spodoptera litura*, *Sitobion avenae*, and *Spodoptera exigua*, it was found that the generation cycle was related to heavy metal concentration, with the low concentration prolonged and the high concentration shortened, and the intrinsic growth rate and net reproductive rate decreased significantly [29,30,31]. Heavy metal pollution also affected the life parameters and behavior of phytophagous insects. *Brevicoryne brassicae* fed on plants that accumulate heavy metals Cd, Zn, and Cu, resulting in developmental asymmetries [32,33]. Under heavy metal stress, the mortality rate of *Sitobion avenae* increased and their reproductive capacity decreased [34]. In our previous experiments, we found that zinc stress had a negative effect on the fertility of aphids and ladybugs [35,36]. In order to alleviate the poisoning symptoms of heavy metals and the damage of heavy metals to the body, insects usually deposit excessive ingested heavy metals in organs with digestive, storage, or secretory functions, such as the digestive tract, fat body, malpighian tubules, exocuticle, and reproductive organs [37]. Excreting feces and molting are other common and effective ways for insects to reduce heavy metal toxicity [37]. Aphids can excrete a small number of heavy metals by secreting honeydew [23,38,39]. For example, aphids dealt with Cd and Cu in different ways. Cadmium accumulated within the body and was rarely excreted through honeydew, but copper was mainly excreted through the honeydew and concentrations in body tissues did not increase [40]. Furthermore, the energy metabolism and innate immune systems of insects play a key role in overcoming heavy metal stress [41].

*Vicia faba* L. is widely planted in China and it is the third most important winter edible soybean crop in the world. During its growth process, it is susceptible to the threat of pests, mainly including the piercing–sucking insects of *Acyrthosiphon pisum* and *Megoura crassicauda* [42]. Infestations weaken plants directly by feeding, and indirectly, by the transmission of yield-affecting plant viruses [43]. In insect hemolymph, 80–90% of the sugars are trehalose, so it is called “blood sugar” [44]. At present, trehalase (TRE) is the only enzyme known to decompose trehalose, which exists in insects in two types: free TRE1 and membrane-bound TRE2, with different functions [45]. Trehalose is not only an energy storage substance that provides energy for life activities, but also an important protective factor that helps organisms resist stress such as dryness, high humidity, low temperature, and oxidation [46]. For example, in a dry environment, the activity of *TPS* in *Drosophila melanogaster* increased while the activity of *TRE* decreased, resulting in an increase in trehalose content in the body [47]. For the *Ostrinia nubilalis*, glycerol and trehalose are the most abundant cryoprotectants in diapause larvae [48]. Reproduction is also an important life process of insects, vitellin (Vn), the main component of livetin, is mediated by vitellogenin (Vg) [49]. It provides nutrients and energy for egg maturation and embryo development, so it is of great significance for the reproduction of most oviparous animals. Current studies have conducted extensive studies on the metabolism and metabolic regulation of trehalose in insects [50], but there are few studies on the effects of heavy metal treatment on trehalose metabolism and other physiological responses of insects. In this study, we aimed to investigate the toxic effects of heavy metal cadmium on insects and the adaptation mechanisms of insects to extreme environments. To achieve this goal, we chose *Megoura crassicauda* as the research object and used the “soil—*Vicia faba* L.—*Megoura crassicauda*” system as a model to explore the bioaccumulation of heavy metal Cd in soil along the food chain, as well as the effects of feeding on Cd contaminated broad beans on aphid growth and development, trehalose metabolism, and reproductive ability.

## 2. Materials and Methods

### 2.1. Test Plant and Insect Source

The model plant used in this study was the broad bean (*V. faba* L.), and the insect source was *M. crassicauda*. Temperature, humidity, and photoperiod were artificially controlled and maintained as follows: temperature 19 ± 1 °C, humidity 70 ± 5%, photoperiod 14L:10D.

### 2.2. Experimental Design

According to Wang et al. [22], Cd^2+^ solutions of 3.125, 6.25, 12.5, 25, and 50 mg/L were set as experimental groups, which were labeled as groups T1, T2, T3, T4, and T5, respectively. Different concentrations of Cd^2+^ solutions were prepared using cadmium chloride as the raw material. The broad bean seeds were soaked for 24 h and then planted in the soil (the volume of nutrient soil: vermiculite: perlite = 12:4:2). Then, based on the growth requirements of broad beans, 400 mL of the corresponding concentration of Cd^2+^ solution was poured every 3 days. Seeds were soaked in tap water (0 mg/L) or watered with tap water as the control, T0 group.

Based on the growth of broad beans, it was determined that on the 10th day of planting broad beans into the soil, the untreated heavy metal adult aphids should be transferred to the broad bean seedlings. After 10 days of infection, the collected aphids (set as the first generation F1) were transferred to the new broad bean seedlings treated with the new Cd^2+^ solution. After the first generation of adult aphids was infected for 10 days, the adult aphids produced in the first generation (the second generation F2) were collected and transferred to the new broad bean seedlings treated with the new corresponding concentration. And so on, for continuous infection. The conditions under which aphids were reared post-Cd exposure were the same as Section 2.1.

### 2.3. Collection of Experimental Materials

The broad bean seeds were soaked in Cd^2+^ solutions of different concentrations and tap water for 24 h. On the 25th day of planting broad beans, during which aphids were transferred to broad bean seedlings on the 10th day, we collected roots, stems, and leaves. The roots were collected by flushing the soil attached to the surface with running water. The five batches of aphids were as follows: the first batch of aphids (F1) referred to all breeding aphids from the first transfer of uninfected adults to broad bean seedlings treated with different concentrations of cadmium until the 25th day of planting of broad beans into the soil. The second batch of aphids (F2) referred to all breeding aphids of the first generation of the broad bean seedlings treated with the corresponding concentration of cadmium ions until the 25th day of planting the broad beans into the soil; F3, F4, F5, and so on. All the above experimental materials were collected, dried, and ground into powder for the determination of heavy metal content.

### 2.4. Determination of Heavy Metal Contents

The Cd content of the samples was determined by inductively coupled plasma mass spectrometry (ICP-MS). The method for determination of Cd content in broad beans seeds, roots, stems and leaves and aphids was as follows: 0.50 g of samples were weighed and placed in a digestion tank. The samples were then placed in 4 mL of nitric acid, 80 °C pre-oxidation for 1 h, and then added into a microwave digestion program for digestion. Subsequently, the samples were transferred to a 50 mL volumetric flask containing raw water to maintain a constant volume, and then the ICP-MS was performed on a machine. Biological replicates were performed three times for each experiment.

### 2.5. cDNA First Strand Synthesis of Aphids and Real-Time Fluorescence Quantitative PCR (qRT-PCR)

From the first generation to the fifth generation, 10 adult aphids per biological replicate were collected and three biological replicates were performed. According to the manufacturer’s instructions, total RNA was extracted from *Megoura crassicauda* using the RNAiso Plus kit (Invitrogen, Carlsbad, CA, USA). Subsequently, 1% agarose gel was used to detect RNA integrity, and NanoDrop™ 2000 (Waltham, MA, USA) was used to determine the concentration and purity of the extracted RNA. A PrimeScript™ RT Reagent Kit with gDNA Eraser (Takara, Kyoto, Japan) was used for reverse transcription of the cDNA. Then, the qRT-PCR reaction was performed. β-actin was an internal control, and the primer sequences are shown in (Table S1). The qRT-PCR data were analyzed using the 2^−ΔΔCT^ method [51]. Note: Table S1 in the Supplementary Document.

### 2.6. Determination of Carbohydrate Content and Trehalase Activity

In excess of fifteen adult aphids (each generation, from F1 to F5) were placed in a 1.5 mL centrifuge tube. The adult aphids were homogenized. They were ground in 200 μL phosphate-buffered saline (PBS) and treated with sonication. Then, 800 μL PBS was added. After centrifuging at 4 °C and 1000× *g* for 20 min, 350 μL of supernatant was used to detect the content of trehalose, glycogen, and protein. The other part of the supernatant was centrifuged at 4 °C, 20,800× *g* for 60 min, and 300 μL of supernatant was used to measure protein and glucose content as well as soluble trehalase (TRE1) activity. Next, 300 μL PBS solution was added to the original centrifuge tube and mixed well to prepare a suspension, which was used to detect the protein and glucose content as well as membrane-bound trehalase (TRE2) activity. The anthrone method was used to detect the trehalose content. Furthermore, a Glucose (GO) Assay Kit (GAGO20, Sigma, St. Louis, MO, USA) was used to measure the glucose and glycogen content, as well as the activity of trehalase. The activity of trehalase depends on the amount of trehalose that can be hydrolyzed by trehalase. The protein content was detected by following the instructions provided in the BCA Protein Assay Kit (P0012, Beyotime, Haimen, China). Three repetitions were performed for each treatment (*n* = 3).

### 2.7. Determination of the Number of Offspring Produced by Female Aphids

On the 10th day of planting broad beans in soil, the raised adult aphids that were not infected by heavy metals were transferred to the broad bean seedlings. After 10 days of infection, the collected aphids (set as the first generation F1) were transferred to the broad bean seedlings treated with the new Cd^2+^ solutions, and the aphid production was counted every 24 h, for a total of 7 days. After the first generation of adult aphids were infected for 10 days, the adult aphids produced in the first generation (the second generation F2) were collected and transferred to the broad bean seedlings treated with the new Cd^2+^ solution, and the total number of aphids produced in 7 days was counted, and so on. The total number of aphids produced in 7 days of the fifth generation was detected. Eight biological replicates were performed for each group.

### 2.8. Data Analysis

The Tukey method in the one-way ANOVA of IBM SPSS Statistics 20 was used to analyze the significance of the data. Finally, GraphPad Prism version 8.4.0 was used to draw the bar chart. The results in the graph are represented by mean ± standard deviation (mean ± SD) or mean ± standard error (mean ± SE), and different letters in the figure indicate significant differences between groups (*p* < 0.05).

## 3. Results

### 3.1. Heavy Metal Content in Broad Beans and Aphids

**Figure 1:**
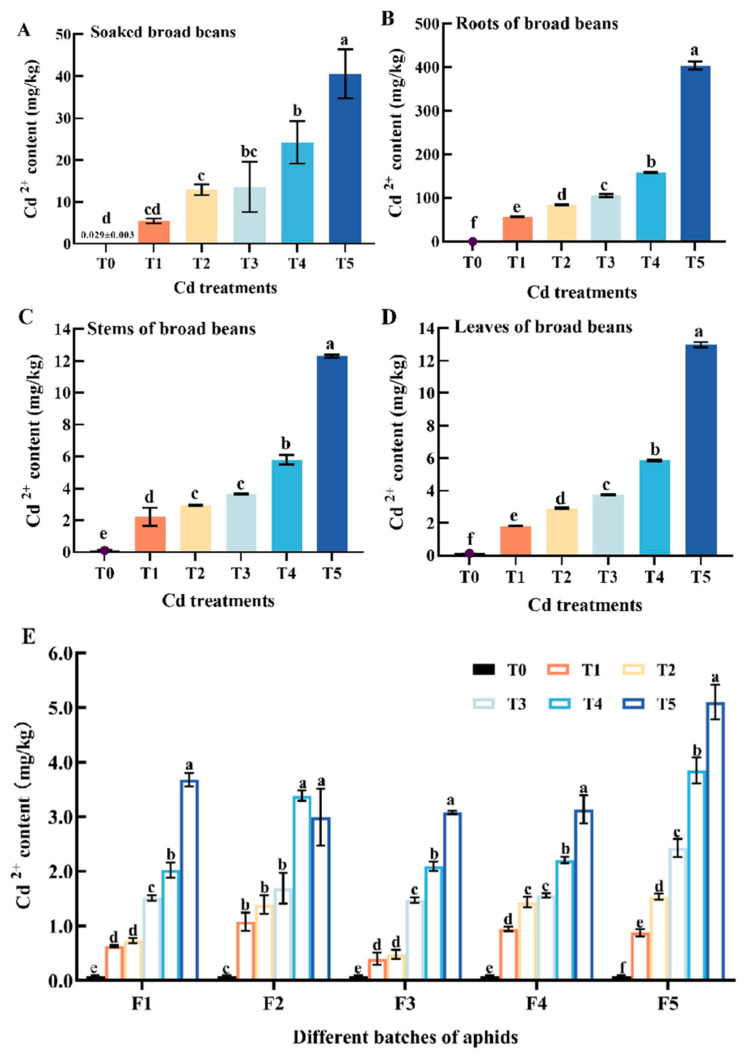
Cd^2+^ content in soaked seeds (**A**), roots (**B**), stems (**C**), and leaves (**D**) of broad beans and five batches of aphids (**E**). Bars represent means (±SD) of three replicate experiments. Bars with different letters indicate significant differences (Tukey method, *p* < 0.05). Note: Tukey method analysis was performed on different groups of aphids from the same batch, with different letters indicating significant differences between the two.

The results show that, compared with the control group, Cd^2+^ content in the seeds, roots, stems, and leaves of broad beans in each Cd^2+^ solution treatment group were increased, and the content increased with the increase of treatment concentration (Figure 1A–D). Cd^2+^ content in broad bean seeds in T2, T3, T4, and T5 treatment groups were significantly higher than that in the T0 group (0.029 ± 0.003 mg/kg) (*p* < 0.05) (Figure 1A). The Cd^2+^ content in the root and the above-ground parts of broad beans in the Cd^2+^ solution treatment group were significantly higher than that in the control group (*p* < 0.05), and the cadmium accumulation in the root was higher than that in the above-ground part (Figure 1B–D). It was indicated that the heavy metal Cd had accumulated in the roots, stems, and leaves of broad beans. The Cd^2+^ content in aphids in each treatment group was higher than that in the control group (Figure 1E). Generally, in F1–F5, the content of Cd in each batch of aphids treated with Cd^2+^ solutions were higher than that in the T0 group (0.081 ± 0.014 mg/kg) (Figure 1E). Further analysis showed that the heavy metal Cd content in each batch of aphids treated with the same concentration was different (Figure 1E).

### 3.2. Changes in the Carbohydrate Content of Adult Aphid in Five Successive Generations

**Figure 2:**
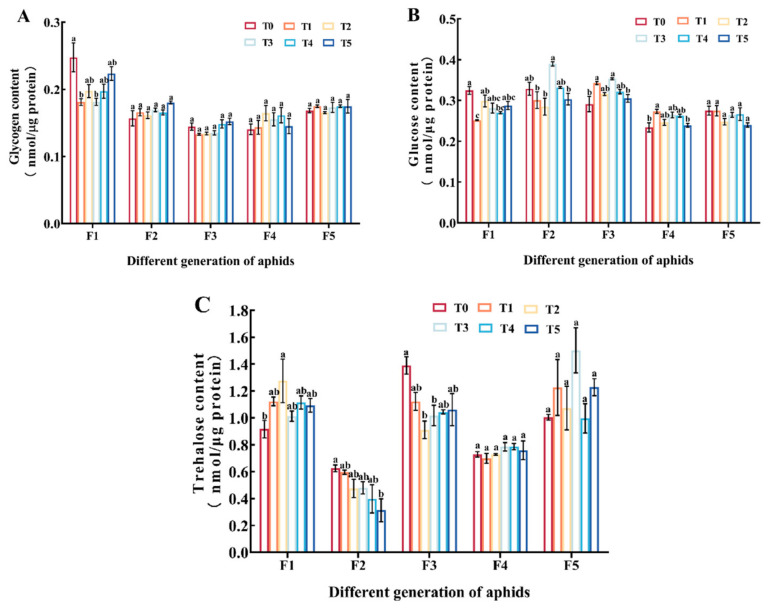
Contents of glycogen (**A**), glucose (**B**), and trehalose (**C**) in different groups of aphids. Bars represent means (±SE) of three replicate experiments. Bars with different letters indicate significant differences (Tukey method, *p* < 0.05). Note: Tukey method analysis was performed on different groups of aphids from the same batch, with different letters indicating significant differences between the two.

In F1, the glycogen content in the Cd treatment group was lower than that in the control group, and the contents of T1 and T3 groups were significantly reduced (*p* < 0.05); however, there was no significant change in glycogen content among F2 to F5 (*p* > 0.05) (Figure 2A). The glucose results showed that in F1, the contents of the T1 and T4 groups were significantly lower than that of the T0 group (*p* < 0.05); in F2, the T3 group had the highest content, followed by the T0 and T4 groups, and the remaining three treatment groups had the lowest content; in F3, the contents of the T1 and T3 groups were significantly higher than that of the T0 and T5 groups (*p* < 0.05); in F4, compared with the control group, the content of the T1 group significantly increased (*p* < 0.05); in F5, there was no significant difference in the content of each group (*p* > 0.05) (Figure 2B). Compared with the control group, F1 treated with Cd resulted in an increase in trehalose content, with a significant change observed in the T2 group (*p* < 0.05); F2 and F3 treated with Cd resulted in a decrease in trehalose content; there was no significant difference in trehalose content between F4 and F5 in each group (*p* > 0.05) (Figure 2C).

### 3.3. Activity Changes of Two Trehalases of Adult Aphid in Five Successive Generations

**Figure 3:**
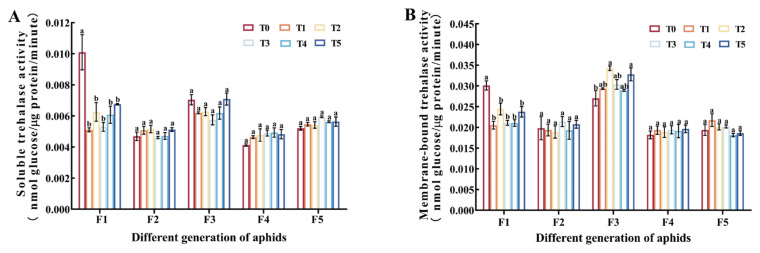
Changes in the activity of two trehalase enzymes in adult aphids of different generations under different Cd concentrations. (**A**) Soluble trehalase activity. (**B**) Membrane-bound trehalase activity. Bars represent means (±SE) of three replicate experiments. Three biological replicates were performed on 10 adult aphids of *M. crassicauda* in each treatment. Bars with different letters indicate significant differences (Tukey method, *p* < 0.05). Note: Tukey method analysis was performed on different groups of aphids from the same batch, with different letters indicating significant differences between the two.

Compared with the control group, the activities of two types of trehalase in F1 treated with Cd were significantly inhibited (*p* < 0.05); the activities of two types of trehalase in F2, F4, and F5 treated with Cd showed no significant changes compared to the control group (*p* > 0.05); there was no significant difference in the activity of soluble trehalose (TRE1) in F3 among the groups (*p* > 0.05), while treatment with 6.25 and 50 mg/L Cd^2+^ solutions (T2 and T5 groups) resulted in a significant increase in the activity of membrane-bound trehalose (TRE2) in adult aphids (*p* < 0.05) (Figure 3).

### 3.4. Changes in Expression Levels of TRE and TPS of Adult Aphids in Five Successive Generations

**Figure 4:**
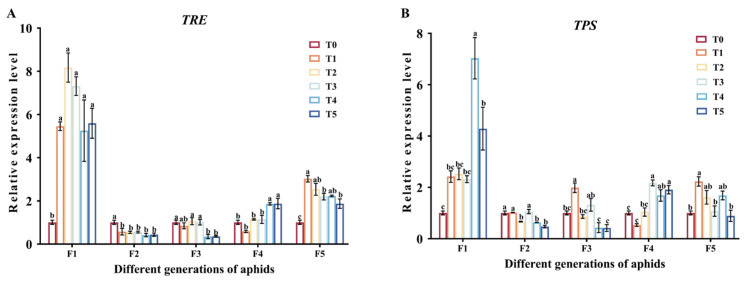
Relative expression levels of trehalase and trehalose-6-phosphate synthase genes in adult aphids of different generations under different cadmium concentrations. (**A**) Trehalase gene, *TRE*. (**B**) Trehalose-6-phosphate synthase gene, *TPS*. Three biological replicates were performed on 10 adult aphids of *M. crassicauda* in each treatment. Bars represent means (±SE) of three replicate experiments. Bars with different letters indicate significant differences (Tukey method, *p* < 0.05). Note: Tukey method analysis was performed on different groups of aphids from the same batch, with different letters indicating significant differences between the two.

In F1, the expression levels of *TRE* and *TPS* in the Cd treatment groups were all significantly higher than those in the control group (*p* < 0.05) (Figure 4A,B). The expression levels of *TRE* in F5 aphids treated with Cd were significantly higher than those in the T0 group (*p* < 0.05), while the expression levels of *TRE* in F2 aphids treated with Cd were significantly lower than those in the T0 group (*p* < 0.05) (Figure 4A). The expression levels of *TRE* in the F3 treated with Cd at concentrations of 25 mg/L and 50 mg/L (T4 and T5 groups) were significantly lower than those in the control group (*p* < 0.05),while in F3, the expressions of *TRE* in these two groups were significantly higher than those in the control group (*p* < 0.05) (Figure 4A). The *TPS* expression levels of F3 and F5 aphids in the T1 group were significantly higher than those in the T0 group (*p* < 0.05) (Figure 4B).

### 3.5. Relative Changes in Vg Levels of Adult Aphid and the Number of Offspring Produced by Female Aphids in Five Successive Generations

**Figure 5:**
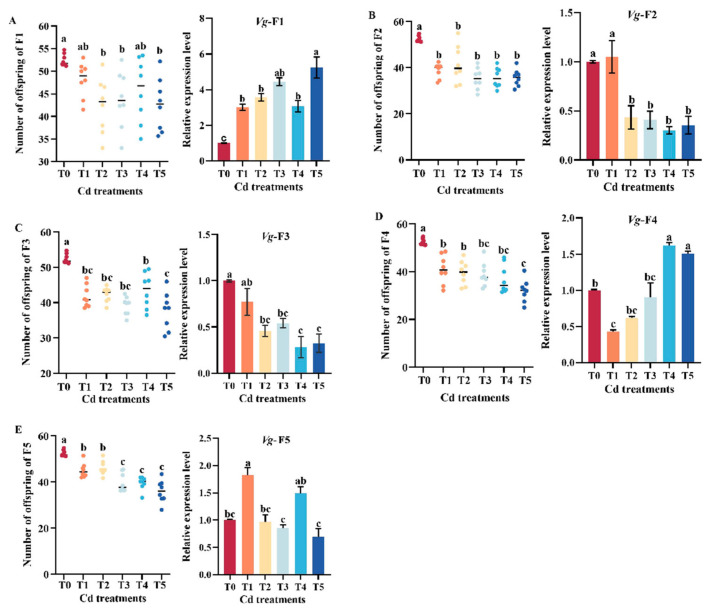
Changes in the number of offspring and vitellogenin gene expression levels of adult aphids from different generations under different Cd concentrations. The number of offspring produced by female aphids from the first generation within 7 days and the expression level of vitellogenin (*Vg*) gene in the aphid on the first day of aphid production statistics (**A**). The number of offspring produced by female aphids from the second generation within 7 days and the expression level of vitellogenin (*Vg*) gene in the aphid on the first day of aphid production statistics (**B**). The number of offspring produced by female aphids from the third generation within 7 days and the expression level of vitellogenin (*Vg*) gene in the aphid on the first day of aphid production statistics (**C**). The number of offspring produced by female aphids from the fourth generation within 7 days and the expression level of vitellogenin (*Vg*) gene in the aphid on the first day of aphid production statistics (**D**). The number of offspring produced by female aphids from the fifth generation within 7 days and the expression level of vitellogenin (*Vg*) gene in the aphid on the first day of aphid production statistics (**E**). Three biological replicates were performed on 8 adult aphids of *M. crassicauda* in each treatment. Bars represent means (±SE) of three replicate experiments. Bars with different letters indicate significant differences (Tukey method, *p* < 0.05). Note: Tukey method analysis was performed on different groups of aphids from the same batch, with different letters indicating significant differences between the two.

Compared with the T0 group, the relative expression level of *Vg* in F1 was significantly increased (*p* < 0.05), while there was a decreasing trend in F2 and F3 (Figure 5A–C). In the F1 female aphids, the number of offspring produced by aphids in the Cd treatment group was lower than that in the T0 group, and there were significant differences between the T2, T3 and T5 groups and the T0 group (*p* < 0.05) (Figure 5A). The numbers of offspring produced by aphids in the F2–F5 female aphids were significantly lower in the Cd treatment groups than in the T0 group (*p* < 0.05) (Figure 5B–E). The above results suggest that the reproductive ability of aphids was affected by Cd pollution and expressed inhibition (Figure 5).

## 4. Discussion

Soil heavy metal pollution is a serious environmental problem that has attracted human attention. Heavy metals in soil or water can be transferred to plants, and their accumulation increased in a dose-dependent manner. Fly ash contained various potentially harmful heavy metals; when mixed with soil, it was found that the absorption of Cd, Pb, and Zn in soil by the roots of *Brassica juncea* increased [38]. Having added different doses of Pb or Cd to the soil, the heavy metal content in *Vicia faba* L. increased [23,39]. This study found that *Vicia faba* L. could directly absorb Cd elements from Cd^2+^ solutions or absorb them through their roots and transport them to the overground parts, namely stems and leaves (Figure 1A–D). There were differences in Cd absorption and accumulation capacity in different parts of plants, which were generally manifested as roots > stems > leaves [52]. Heavy metals in soil are usually absorbed by plant roots in bioavailable form through extracellular and isoplasmic pathways [53,54]. In this study, we found that the Cd absorption and accumulation capacity of broad bean root was the strongest (Figure 1A–C). This might be due to the different absorption pathways of heavy metals in various parts [23,39] of plants, with roots often retaining most of the metals to protect above-ground parts. Transfer coefficients for heavy metals between roots and above-ground parts are usually below 1 [55]. Relevant studies found that Cd could be transferred along the food chain composed of soil-*Populus alba berolinensis* seedlings-*gypsy* moth larvae, and the transfer of Cd between *Populus alba berolinensis* seedlings and *gypsy* moth larvae exhibited a biomagnification effect [56,57,58]. In this study, we found that aphids fed Cd-contaminated broad beans resulted in increased Cd content in the body, and increased with the increase in Cd^2+^ solution concentration (Figure 1E). Here, aphids are exposed to cadmium mainly through the “soil-plant-insect” food chain. Feeding patterns and the content of heavy metals in plants would affect the accumulation of heavy metals in herbivorous insects [55]. In addition, we found that the fifth generation of aphids accumulated more cadmium content in their bodies compared to the first generation (Figure 1E). Excretion is one of the important ways to reduce the accumulation of heavy metals in insects [59]. We speculated that during F1, aphids might excrete some cadmium from their bodies, leaving some residual cadmium in their bodies, which is transmitted to their offspring through reproduction and other means. Additionally, heavy metal pollution might affect the genetic structure of aphids. More relevant mechanisms need to be further studied.

Trehalose plays a crucial regulatory role in various physiological activities of insects. Trehalose is the main energy source of plant-eating insects, and it also acts as a stress protector [60]. Among the reported trehalose biosynthesis pathways in insects, trehalose is synthesized mainly through the trehalose-6-phosphate synthase (TPS)/trehalose-6-phosphate phosphatase (TPP) pathway or TPS pathway [61]. Trehalase (TRE) catalytic trehalose is decomposed into two glucose molecules, in the process to release the large amount of energy is used in a variety of physiological activities [62]. TPS is an important enzyme involved in trehalose synthesis [63]. In order to resist the toxic risk brought by heavy metals, organisms will respond to heavy metal stress by regulating some physiological and biochemical metabolism in their bodies [37]. After feeding on Cd-accumulated *Hyphantria cunea* larvae, the glucose content in *Arma chinensis* nymphs decreased evidently [21]. It was found that trehalose metabolism of *Aedes albopictus* was affected under acute Cd stress [64]. In addition, trehalose content of *Aedes albopictus* increased under long-term Cd stress [50], while glucose content and trehalase activity decreased. The results of this study also confirmed this, and it was found that with the increase in stress generations, the metabolism of trehalose in aphids changed (Figure 2 and Figure 3). Cd pollution had a significant impact on the first generation of aphids. Aphids could increase the synthesis of trehalose by consuming glycogen, inhibiting the activity of trehalase (Figure 2 and Figure 3). This may be due to the stress response of aphids when initially exposed to Cd stress, leading to an increase in trehalose synthesis as a protective agent for themselves. Comparable finds were noted in *Agrotis ipsilon* larvae; the amount of glycogen decreased dramatically in the presence of heavy metal contamination [65]. This result could be explained as a decrease in energy storage and an increase in metabolic rate after cadmium exposure [66]. In F1, TPS expression was significantly upregulated in the cadmium treated group (Figure 4B). The increase in relative expression levels of *TPS* is consistent with changes in trehalose content, indicating that *TPS* increased trehalose content through its own upregulation of expression. In the cadmium treatment group, the activity of TRE1 and TRE2 decreased (Figure 3). It is worth noting that the relative expression level of *TRE* was opposite to the change in TRE enzyme activity (Figure 3 and Figure 4A). We speculated that this was due to the increase in trehalose levels and negative feedback regulation to maintain homeostasis in the body. The trehalose content in F2 and F3 decreased, and it was speculated that trehalose decomposition was increased to resist Cd stress. The effects of Cd pollution on F4 and F5 were very small, indicating that the adaptability of the aphids to stress increased with the increase in stress generations (Figure 2 and Figure 3). Resistance to heavy metals is a physiological process that requires energy, and heavy metal resistance usually leads to energy metabolism disorders in insects [67]. In addition, in our previous research, we also found that insects could resist heavy metal stress by regulating trehalose metabolism [68]. In summary, it indicated that insects could resist Cd stress by regulating trehalose metabolism, and heavy metals could affect the energy metabolism of insects and then affect the content of energy substances in the bodies of insects, and the specific mechanism needs to be further studied.

Heavy metal pollution also has a certain effect on biological fertility. Heavy metals can delay the synthesis of vitellinogen polypeptide, thereby delaying ovarian maturation and inhibiting vitellogenesis [69]. In our previous study, we found that the expression of *Vg1*, *Vg2*, and *VgR* in female adults were inhibited under zinc stress, resulting in a decrease in egg production and hatching rate [36]. In this study, it was found that the relative expression of *Vg* in aphids in F2 and F3 showed a downward trend compared with the control group (Figure 5B,C), which indicated that down-regulation expression of *Vg* content inhibited the reproduction of female aphids. But the relative expression of Vg in F1 was significantly higher than that in the control group (Figure 5A). Further research is needed on this phenomenon and its mechanism. In addition, it was found that when feeding on *Aleyrodidae* contaminated with Pb, Ni, or Cd, the preoviposition period of *Cryptolaemus montrouzieri* was longer than that of the control group, and egg production was less than that of the control group (Sang et al., 2018) [70]. Cd significantly prolonged the mating latency of female *Drosophila melanogaster* and decreased egg production [71]. It was also found that the fecundity of female adult *Spodoptera litura* was inhibited under high concentration of Zn stress [72]. The results of this study are similar. When feeding on Cd-contaminated broad beans, the aphid productions for five successive generations were all lower than control groups (Figure 5), indicating that the reproductive ability of aphids was inhibited by Cd pollution. But it has been found in other studies that the reproduction of *Myzus persicae* was not influenced by the presence of Cd and its chelator [73]. In plant experiments, other treated aphid species, such as *Brevicoryne brassicae* and *Acyrthosiphon pisum*, showed a decrease in reproductive ability when reared on plants containing cadmium [74,75]. The relevant mechanism needs further study.

## 5. Conclusions

In summary, Cd could be transmitted and accumulated between broad beans and aphids through the food chain, and the accumulation amount was related to the concentration in the environment and it varied from generation to generation. Cd could affect the energy metabolism of aphids, and then changed the content of energy substances in the body. In addition, the reproduction of female aphids was inhibited. Our research achievements provide a theoretical basis for studying the toxic effects of heavy metals on insects and the adaptation mechanisms of insects to heavy metal stress, and also lay a certain foundation for explaining how such an accumulation may have implications for ecosystem health, agricultural pest control, or even human exposure risks.

## Data Availability

The data presented in this study are available on request.

## Supplementary Materials

The following supporting information can be downloaded at: https://www.mdpi.com/article/10.3390/insects15120999/s1, Table S1: The primers for real-time fluorescence quantitative PCR.
