# Supplementary material for: The Stress Response of Aphids to the Accumulation of Heavy Metals Along Vicia faba L. Under Cadmium Treatment
Source: Insects. 2024 Dec 16;15(12):999. doi: 10.3390/insects15120999 (PMC11678626; doi:10.3390/insects15120999)
Supplement: Supplementary file 1 [file insects-15-00999-s001.zip › insects-3326450-supplementary.pdf]

Table S1 The primers for real-time fluorescence quantitative PCR

Gene name	Forward primer (5′-3′)	Reverse primer (5′-3′)
Actin	GATCATTGCCCCACCAGAAC	TTTACGGTGGACAATGCCTG
TPS	CGTGGACAGGCTAGACTACA	CAGCTCAGTCTCGTCCTTGA
TRE	TGGCAAGATACTACGCACCA	ATCAGCCAATACCCCACGAT
Vg	GCATTAGCCACTATGTTTCA	CGTATTGCTCCATTGTTGT
